# Skin Deep: A Case Report of Erythema Nodosum Induced by Dupilumab Therapy

**DOI:** 10.7759/cureus.115399

**Published:** 2026-08-29

**Authors:** Nisat Tasnim, Jerrin Bawa, Rasha Alhashimi, Erika Malana, Jobanjit Kaur, Nailah Sayeed, Aizaaz Khan

**Affiliations:** 1 Internal Medicine, Flushing Hospital Medical Center, New York, USA; 2 Dermatology, Larkin Community Hospital, Miami, USA; 3 Pulmonary and Critical Care, Flushing Hospital Medical Center, New York, USA

**Keywords:** dupilumab adverse reaction, erythema nodosum, interleukin-13, interleukin 4 (il-4), panniculitis

## Abstract

Dupilumab is a fully human monoclonal antibody that inhibits interleukin (IL)-4 and IL-13 signaling, thereby attenuating type 2 inflammatory pathways. It is currently approved for the treatment of severe eosinophilic asthma, atopic dermatitis, chronic rhinosinusitis with nasal polyps, eosinophilic esophagitis, and other allergic conditions. Erythema nodosum (EN) is the most common form of panniculitis, characterized by tender, erythematous subcutaneous nodules typically distributed over the extensor surfaces of the lower extremities. Drug-induced EN represents a minority of all EN cases and has been rarely described in association with biologic therapies including anti-IL-4/13 and anti-IL-5 agents. Notably, dupilumab-associated EN is exceedingly rare, and this report describes a case of clinically suspected dupilumab-associated EN occurring in a patient with severe eosinophilic asthma in the setting of suppressed peripheral blood eosinophils following a treatment interruption.

We present the case of a 68-year-old woman with severe eosinophilic asthma who developed clinically suspected EN shortly after initiation of dupilumab therapy and a subsequent two-month treatment interruption. She presented with painful bilateral lower extremity and upper arm nodules following her fifth injection. Notably, her peripheral blood eosinophil count, which had been persistently elevated for more than one year before dupilumab initiation, transiently rebounded during the treatment gap but was adequately suppressed (0.2 × 10^3^/μL) at the time of presentation, reflecting the combined immunomodulatory effects of dupilumab and recent short-term systemic corticosteroid therapy. The diagnosis of clinically suspected dupilumab-associated EN was supported by characteristic clinical findings, a suggestive temporal association with drug reinitiation, and the systematic exclusion of alternative etiologies. Symptoms resolved following discontinuation of dupilumab and initiation of supportive management.

This case demonstrates that clinically suspected dupilumab-associated EN may occur in the absence of peripheral blood eosinophilia. Clinicians should maintain awareness that normal or suppressed peripheral eosinophil counts do not preclude the development of paradoxical immune-mediated cutaneous reactions during biologic therapy.

## Introduction

Dupilumab (Dupixent®) is a fully human immunoglobulin G4 monoclonal antibody that selectively targets the interleukin-4 receptor alpha (IL-4Rα) subunit, a shared structural component of both the type I and type II interleukin (IL)-4 receptor complexes, thereby simultaneously inhibiting downstream signaling of IL-4 and IL-13, the principal cytokines driving type 2 (T-helper (TH)2)-mediated inflammation [1,2]. Since its initial Food and Drug Administration approval for moderate-to-severe atopic dermatitis in 2017, dupilumab has received regulatory approval for multiple additional type 2 inflammatory conditions, including moderate-to-severe eosinophilic asthma, chronic rhinosinusitis with nasal polyps, eosinophilic esophagitis, prurigo nodularis, and bullous pemphigoid [1]. In the context of eosinophilic asthma, IL-4 and IL-13 promote eosinophil recruitment, immunoglobulin E synthesis, mucus hypersecretion, and airway hyperreactivity; clinical trials have demonstrated that dupilumab significantly reduces severe asthma exacerbations, improves lung function, and decreases oral corticosteroid dependence in this population [1-3].

Despite its established efficacy and generally favorable safety profile, the expanding clinical use of dupilumab has been accompanied by increasing recognition of paradoxical cutaneous adverse reactions. The suppression of TH2-dominant signaling has been reported to disinhibit TH1 and TH17 immune axes, pathways normally held in check by IL-4 and IL-13, resulting in inflammatory cutaneous conditions such as psoriasiform eruptions, lichenoid drug reactions, and, rarely, erythema nodosum (EN) [4,5]. Paradoxical cutaneous reactions have also been documented with other TH2-targeted biologics used in eosinophilic asthma, including anti-IL-5 agents (mepolizumab, reslizumab) and anti-IL-5 receptor alpha monoclonal antibodies (benralizumab), highlighting a broader class-related phenomenon of immune axis rebalancing during targeted cytokine inhibition.

EN is the most common form of panniculitis, characterized by tender, bilateral, erythematous subcutaneous nodules typically localized to the anterior and lateral surfaces of the lower extremities [6]. Histopathologically, EN is characterized by septal panniculitis without vasculitis, with an early neutrophilic infiltrate that transitions to a lymphohistiocytic infiltrate with granuloma formation in later stages [6,7]. Recognized etiologies include infections, systemic inflammatory diseases, medications, malignancy, and pregnancy; in approximately 30%-50% of cases, no identifiable cause is found [6]. Drug-induced EN accounts for approximately 3%-10% of all EN cases, with implicated agents including oral contraceptives, sulfonamides, penicillin, and, more recently, biologic therapies such as tumor necrosis factor alpha (TNF-α) inhibitors and immune checkpoint inhibitors [6,7].

Among the exceedingly rare reports of dupilumab-associated EN in asthma, this case contributes important clinical data regarding EN onset in the presence of pharmacologically suppressed peripheral blood eosinophils, raising hypotheses regarding potential eosinophil-independent or localized tissue-driven immune mechanisms [8].

## Case presentation

A 68-year-old woman presented to the outpatient clinic with a chief complaint of erythematous, painful, throbbing, bilateral upper and lower extremity cutaneous lesions of acute onset. Her past medical history includes severe persistent asthma with an eosinophilic phenotype, aspirin allergy, and nasal polyps (aspirin-exacerbated respiratory disease), type 2 diabetes mellitus, osteoporosis, gastroesophageal reflux disease, and anxiety.

The patient had been initiated on subcutaneous dupilumab (200 mg every two weeks) as add-on therapy for severe persistent eosinophilic asthma approximately five months prior to presentation. Her baseline pretreatment laboratory evaluation demonstrated a persistently elevated peripheral blood absolute eosinophil count (AEC) of 1.1 × 10³/μL, which had been documented for over one year prior to therapy. The total five-month timeline was structured as follows: the patient received three initial biweekly doses (Doses 1-3) over the first month. Thereafter, she experienced a two-month treatment interruption during international travel, during which she missed approximately four consecutive biweekly doses. While traveling, she experienced a mild asthma flare managed with a short five-day course of oral prednisone (20 mg daily), which was completed 20 days before the fourth dupilumab administration.

Upon returning from travel, the patient restarted dupilumab therapy and received Dose 4. Laboratory evaluation at the time of Dose 4 demonstrated a transient rebound of peripheral AEC to 1.1 × 10³/μL, attributable to drug washout during the two-month treatment gap. Two weeks later, she received her fifth dupilumab injection (Dose 5). Exactly five days following Dose 5, cutaneous symptoms acutely manifested, establishing a suggestive temporal association between drug reexposure and lesion onset. Her concurrent daily controller medications included montelukast (10 mg daily), budesonide/formoterol combination inhaler (160/4.5 mcg, two puffs twice daily), and albuterol inhaler as needed.

On physical examination, erythematous, solid, subcutaneous nodules measuring 3-20 cm in diameter were identified on the bilateral extensor surfaces of the lower legs and upper extremities (Figure 1). The lesions were tender to palpation, warm, and associated with a characteristic throbbing discomfort, consistent with the clinical presentation of EN [6,7]. Evaluation by a dermatologist confirmed clinical features characteristic of septal panniculitis. The performance of a skin biopsy was thoroughly discussed with the patient to obtain histopathologic confirmation; however, after weighing the risks and benefits, the patient declined a skin biopsy due to localized tenderness and expressed a preference for noninvasive, conservative monitoring. Consequently, the diagnosis was established as a clinically diagnosed suspected EN.

**Figure 1:**
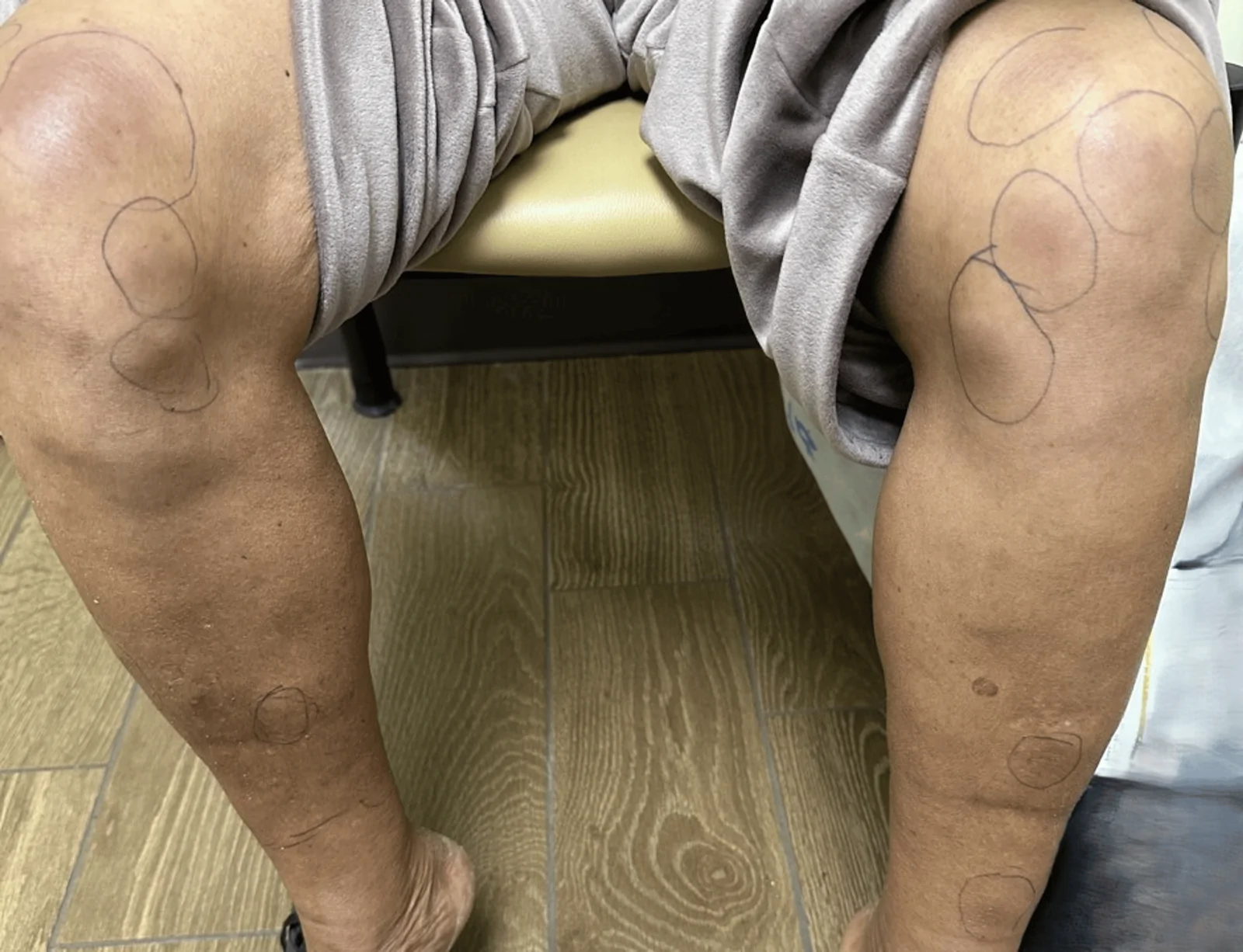
Erythematous, solid, subcutaneous nodules, 3-20 cm in diameter, localized on the bilateral extensor surfaces of the legs, consistent with the clinical presentation of erythema nodosum in the context of dupilumab therapy

At the time of presentation (Dose 5), laboratory evaluation demonstrated that the patient's peripheral blood AEC was suppressed to 0.2 × 10³/μL, attributable to the cumulative immunomodulatory effect of reinitiated dupilumab therapy combined with the lingering suppressive effect of recent oral corticosteroid exposure (Table 1). White blood cell count was elevated at 15.0 × 10³/μL with absolute neutrophilia (10.9 × 10³/μL), which was attributed to systemic corticosteroid-induced neutrophilic demargination combined with the acute-phase inflammatory response accompanying acute EN.

**Table 1:** Serial laboratory results across dupilumab treatment timeline in a patient with severe persistent eosinophilic asthma who developed clinically suspected dupilumab-associated erythema nodosum Empty cells (-) indicate parameters not collected at that time point EN: erythema nodosum; ALT: alanine aminotransferase; AST: aspartate aminotransferase; SGPT: serum glutamic-pyruvic transaminase; CBC: complete blood count

Category/parameter	Unit	Reference range (adult female)	Dupilumab dose 1 (Dupixent 200 mg)	Dupilumab dose 2 (Dupixent 200 mg)	Dupilumab dose 3 (Dupixent 200 mg)	2 Consecutive missing dupilumab doses	Dupilumab dose 4 (Dupixent 200 mg)	Dupilumab dose 5 (Dupixent 200 mg)	EN occurrence
CBC - hemogram									
White blood cells	×10³/µL	4.5-11.0	8.1	4.4	8.1	-	7.9	15	10.4
Red blood cells	×10³/µL	3.90-5.20	4.37	4.01	4.31	-	4.29	4.11	4.23
Hemoglobin	g/dL	12.0-16.0	13.1	12.3	12.8	-	12.6	11.9	12.8
Hematocrit	%	36.0-46.0	40.4	36.7	39.4	-	38.7	37.1	38.8
Mean corpuscular volume	fL	80.0-100.0	92.4	91.7	91.3	-	90.2	91.8	91.8
Mean corpuscular hemoglobin	pg	27.0-33.0	30	30.8	29.8	-	29.3	28.9	30.2
Mean corpuscular hemoglobin concentration	g/dL	32.0-36.0	32.5	33.6	32.6	-	32.5	32	32.9
Red cell distribution width	%	11.5-14.5	14.4	15.2	14.2	-	15.3	15.8	16.4
Mean platelet volume	fL	7.5-12.5	7.4	6.9	7	-	7.8	7	7.3
Platelet count	×10³/µL	150-400	317	426	449	-	310	402	316
Automated differential									
Neutrophils	%	50.0-70.0	48.1	40.1	63.4	-	48.4	72.9	74.6
Lymphocytes	%	20.0-40.0	30.2	38.6	23.6	-	30.3	18.5	14.4
Monocytes	%	2.0-10.0	7.5	10.6	8.5	-	6.8	7.2	7.8
Eosinophils	%	1.0-4.0	13.5	9.5	4.1	-	14.1	1	2.4
Basophils	%	0.0-1.0	0.7	1.2	0.4	-	0.4	0.4	0.8
Absolute neutrophils	×10³/µL	1.8-7.7	2.5	1.8	5.1	-	3.8	10.9	7.8
Absolute lymphocytes	×10³/µL	1.0-4.8	2.5	1.7	1.9	-	2.4	2.8	1.5
Absolute monocytes	×10³/µL	0.1-0.9	0.6	0.5	0.7	-	0.5	1.1	0.8
Absolute eosinophils	×10³/µL	0.0-0.5	1.1	0.4	0.3	-	1.1	0.1	0.2
Absolute basophils	×10³/µL	0.0-0.1	0.1	0.1	0	-	0	0.1	0.1
General chemistry									
Glucose	mg/dL	70-99	89	123	170	-	217	160	131
Urea nitrogen (blood urea nitrogen)	mg/dL	7-25	19	14	15	-	20	17	18
Creatinine	mg/dL	0.50-1.10	0.7	0.8	0.7	-	0.6	0.6	0.6
Sodium	mEq/L	136-145	139	141	134	-	132	139	137
Potassium	mEq/L	3.5-5.1	5.1	5.4	4.6	-	4.5	5.3	5
Chloride	mEq/L	98-107	103	105	102	-	98	104	105
Carbon dioxide	mEq/L	22-29	23	30	25	-	23	26	27
Calcium	mg/dL	8.5-10.5	9.7	9.5	9.5	-	9.7	9.8	9.3
Anion gap	mEq/L	8-16	13	6	7	-	11	9	5
Phosphorus	mg/dL	2.5-4.5	-	-	4.3	-	-	-	-
Protein, total	g/dL	6.3-8.2	7.7	6.8	7.1	-	-	7.3	7.4
Albumin	g/dL	3.5-5.0	4.8	4.2	4.1	-	-	4.3	4.4
Bilirubin, total	mg/dL	0.2-1.2	0.4	0.6	0.3	-	-	0.5	0.4
ALT (SGPT)	U/L	7-35	20	40	21	-	-	24	20
AST	U/L	10-40	27	32	18	-	-	28	34
Alkaline phosphatase	U/L	35-105	59	58	62	-	-	65	56

The differential diagnosis included cellulitis, vasculitis, nodular vasculitis, and drug-induced panniculitis. Alternative etiologies were systematically excluded through clinical history, systems review, and laboratory assessment. To evaluate for underlying secondary etiologies, serial blood cultures were obtained at 24, 48, 72 hours, and five days, demonstrating no growth of pathogenic microorganisms and ruling out systemic bacteremia. Furthermore, chest imaging (chest radiograph) revealed clear lung fields without hilar lymphadenopathy, parenchymal opacities, or infiltrates, rendering active pulmonary tuberculosis, sarcoidosis, and overt intrathoracic malignancy highly unlikely. The diagnosis of clinically suspected dupilumab-induced EN was supported by the compelling temporal relationship between drug readministration and lesion onset. Application of the Naranjo Adverse Drug Reaction (ADR) Probability Scale yielded a total score of 7, corresponding to a probable ADR [9]. Dupilumab was permanently discontinued, and the patient was initiated on supportive management. For concurrent asthma control, she received a standard six-day oral methylprednisolone taper (4 mg Dosepak; 24 mg daily initial dose, decremented by 4 mg daily through Day 6). Follow-up clinical and laboratory evaluations were conducted at two and four weeks after discontinuation. The peripheral AEC was 0.17 × 10³/μL at two weeks and rose to 0.59 × 10³/μL at four weeks. Complete resolution of the cutaneous lesions was documented, with no recurrence observed during subsequent monitoring.

## Discussion

The present case adds to the exceedingly limited literature on dupilumab-associated EN in severe eosinophilic asthma [8]. Causal attribution of EN to dupilumab is suggested by the clear temporal relationship between lesion onset following Dose 5 reexposure and complete resolution after drug discontinuation, a pattern consistent with established criteria for ADRs [9]. Causal attribution of EN to dupilumab was further supported by application of the Naranjo ADR Probability Scale [9]. Relevant criteria met in this case included the existence of a previous report of this reaction (+1), onset of the adverse event following drug administration (+2), clinical improvement after drug discontinuation (+1), absence of identifiable alternative causes (+2), and the presence of objective clinical evidence (+1), yielding a total score of 7. This score corresponds to a probable ADR, providing additional structured support for dupilumab as the causative agent in this case. Following editorial guidelines, the individual 10-question scale is detailed descriptively in text rather than reproduced in tabular format to comply with copyright guidelines.

A critical aspect of this case involves the pharmacokinetic impact of the two-month treatment interruption. Following initial suppression during Doses 1-3, the patient missed approximately four biweekly doses while traveling, allowing drug washout. This washout resulted in a rebound of peripheral AECs back to baseline levels (1.1 × 10³/μL) at Dose 4. Upon drug reinitiation, peripheral eosinophils were resuppressed to 0.2 × 10³/μL by Dose 5. The manifestation of EN five days post-Dose 5 occurred specifically during this secondary phase of peripheral eosinophil suppression. In the previously published case of dupilumab-induced EN by Mustin et al., the onset of EN was accompanied by a marked rise in peripheral blood eosinophils, suggesting an eosinophil-driven process [8]. In contrast, the present case occurred in the setting of suppressed peripheral AEC. However, it is important to emphasize that peripheral blood eosinophil suppression does not establish the absence of localized tissue eosinophilia or local tissue effector cell activation. Biologic blockade of IL-4Rα inhibits IL-4/IL-13-driven tissue recruitment and survival signaling, but localized immune complexes or alternative inflammatory cascades may still operate within subcutaneous septa [4].

The pathogenesis of EN is multifactorial, involving immune complex deposition within subcutaneous venules, neutrophil recruitment driven by proinflammatory cytokines, and TNF-mediated innate immune responses [6]. We hypothesize that targeted blockade of the TH2 axis by dupilumab may paradoxically disinhibit the TH1 and TH17 pathways [4,5]. As conceptualized in Figure 2, upregulation of the TH1/TH17 axis can promote downstream neutrophilic activation, localized TNF production, and immune complex deposition within subcutaneous septa, thereby providing a hypothetical substrate for EN development [4,5]. The observed neutrophilic leukocytosis (10.9 × 10³/μL) at presentation aligns with this neutrophilic septal inflammatory hypothesis and with steroid-induced neutrophilic demargination [10].

**Figure 2:**
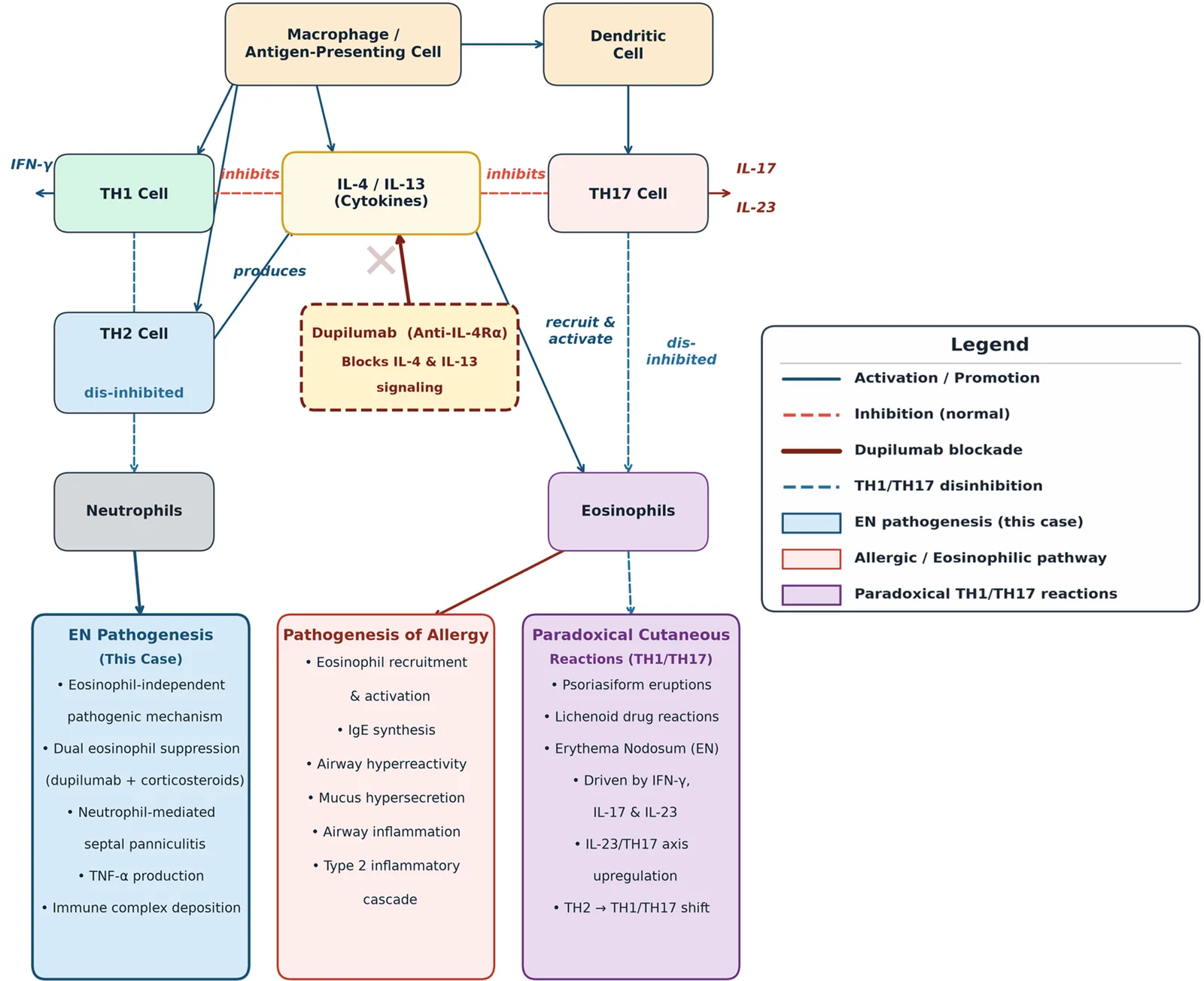
Proposed hypothetical model of dupilumab-associated erythema nodosum pathogenesis Dupilumab blocks IL-4Rα, inhibiting IL-4/IL-13 signaling and suppressing eosinophil-driven type 2 inflammation. Disinhibition of TH1/TH17 pathways fosters paradoxical cutaneous reactions. EN in this case arose via an eosinophil-independent mechanism: neutrophil-mediated septal inflammation, TNF-α production, and immune complex deposition IL-4Rα: interleukin-4 receptor alpha; IL-4: interleukin 4; IL-13: interleukin 13; IL-17: interleukin 17; IL-23: interleukin 23; TH1: T-helper 1; TH2: T-helper 2; TH17: T-helper 17; EN: erythema nodosum; TNF-α: tumor necrosis factor alpha; IFN-γ: interferon-gamma; IgE: immunoglobulin E Image credit: This figure was generated by the author Nisat Tasnim using Microsoft PowerPoint (Office 365; Microsoft Corporation, Redmond, WA)

Clinically, this case suggests that peripheral blood eosinophil counts cannot reliably serve as surrogate markers to exclude the risk of EN or other cutaneous adverse events during dupilumab therapy [11,12]. Clinicians should maintain proactive dermatologic surveillance in patients receiving TH2-targeted biologics regardless of circulating eosinophil numbers [12]. The primary methodological limitation of this case report is the absence of histopathological verification via skin biopsy, as the patient declined invasive testing. Although clinical presentation and expert dermatologic evaluation strongly supported EN, definitive histological differentiation from other forms of panniculitis or vasculitis could not be performed [6]. Additionally, localized tissue cytokine profiling and immunophenotyping were not measured. Finally, the two-month treatment interruption and concurrent short-term corticosteroid use represent confounding variables that influence systemic immune cell counts and drug kinetics [10].

## Conclusions

This case demonstrates that clinically suspected dupilumab-associated EN may occur in the absence of peripheral blood eosinophilia. Early recognition and prompt drug withdrawal remain the mainstays of clinical management, leading to complete symptom resolution in this patient. Although limited by the absence of histopathological confirmation, a Naranjo score of 7 supports dupilumab as a probable causative agent. Further pharmacovigilance reporting and mechanistic studies are warranted to characterize paradoxical immune reactions associated with IL-4/IL-13 blockade.
